# Validation of a locked nucleic acid based wild-type blocking PCR for the detection of *EGFR* exon 18/19 mutations

**DOI:** 10.1186/s13000-015-0293-1

**Published:** 2015-05-29

**Authors:** Liesbet Vliegen, Christophe Dooms, Wim De Kelver, Eric Verbeken, Johan Vansteenkiste, Peter Vandenberghe

**Affiliations:** Center for Human Genetics, University Hospitals Leuven, Herestraat 49,, Leuven, B–3000 Belgium; Respiratory Oncology Unit, University Hospitals Leuven, Herestraat 49, Leuven, B-3000 Belgium; Department of Pathology, University Hospitals Leuven, Herestraat 49, B–3000 Leuven, Belgium

**Keywords:** *EGFR* mutations, Non-small cell lung cancer, LNA, PCR

## Abstract

**Background:**

Treatment decisions in advanced non-small cell lung cancer rely on accurate analysis of the *EGFR* mutation status in small tissue samples. Sanger sequencing of PCR products is unbiased and cheap, but its detection threshold requiring 20 % infiltration by malignant cells is not optimal. Commercial kits, based on quantitative real-time PCR have better detection limits and can detect a wide spectrum of mutations but are considerably more expensive.

**Methods:**

We developed a wild-type blocking PCR for *EGFR* G719A/S/C (exon 18), exon 19 deletions, and exon 20 insertions using locked nucleic acid (LNA) probes. The amplification products of positive reactions were analyzed by Sanger sequencing. We retrospectively validated this assay by comparison of the *EGFR* mutation status as obtained with Fragment Length Analysis and the Therascreen EGFR RGQ PCR kit.

**Results:**

The *EGFR* mutation status for exon 18 and 19 as obtained with the LNA-PCR/sequencing assay correlated adequately with the results obtained by the other independent methods. Due to the lack of structural consistency among the insertions in exon 20, the latter are less amenable for a LNA-PCR design.

**Conclusions:**

The LNA-PCR/sequencing assay presented here is specific, sensitive, and has a low detection threshold. In combination with allele-specific PCR reactions for T790M (exon 20) and L858R (exon 21), a wider scope of *EGFR* mutations can be assessed at a lower cost.

**Virtual slides:**

The virtual slide(s) for this article can be found here: http://www.diagnosticpathology.diagnomx.eu/vs/1272520418142748

## Background

Lung cancer is a leading cause of cancer-related mortality worldwide [1]. Non-small cell lung cancer (NSCLC) accounts for more than 80 % of all lung cancers. About ten percent of NSCLC cancers harbor activating mutations in the Epidermal Growth Factor Receptor (*EGFR*) gene. The most common activating mutations of *EGFR* occur in the intracellular tyrosine kinase domain, i.e. in exons 18–21 [2, 3]. These mutations lead to constitutive activation and convey transforming capacity to the receptor. Deletions in exon 19 and a point mutation in exon 21 (L858R) account for about 90 % of *EGFR* mutations reported to date [4, 5]. The majority (44 to 80 %) of exon 19 deletions are in-frame micro-deletions encompassing amino acids 746–750 (ELREA) [5, 6]. The G719A mutation within exon 18 results in an amino acid substitution at position 719, from a glycine (G) to an alanine (A). Two additional independent mutations have also been described at this position: G719C (Cysteine) and G719S (Serine). Collectively, these point mutations occur with a frequency of approximately 3 % [5]. G719A/S/C, exon 19 deletions, and L858R mutations are associated with sensitivity to *EGFR* tyrosine kinase inhibitors (*EGFR*-TKIs) gefitinib, erlotinib, or afatinib [7–10]. Drug resistance emerges most frequently as a result of a secondary mutation in exon 20 (T790M) [11, 12]. The T790M mutation is almost always observed in conjunction with a sensitivity-conferring mutation. Combinations of in-frame insertions and/or duplications of 3 to 21 base pairs in *EGFR* exon 20 account for another 4 % of *EGFR* mutations. These exon 20 insertions are predominantly clustered between amino acids 767 and 774 and represent a highly heterogeneous family of activating mutations with a variable length, position, and amino acid composition. Most patients with tumors with insertion mutations in exon 20 are less sensitive to TKIs [13–15].

As the majority of NSCLC cases present with advanced stage, their diagnosis is usually based on small biopsy and cytology specimens obtained by bronchoscopic procedures, and not on surgical resection specimens [16, 17]. Analysis of the *EGFR* mutation status on these limited amounts of tissue sample poses a technical challenge, yet is strictly required as the results will determine therapeutic decisions.

Several methodologies are available for *EGFR* mutation testing [16, 18]. One is direct Sanger sequencing of the mutated exons. Sanger sequencing is non-biased and will detect all sequence variants, including rare ones. The major disadvantage is its detection threshold requiring at least a 10 % mutant allele fraction, or at least a 20 % malignant cell fraction. To obviate this, a variety of quantitative real-time PCR (qPCR) methods have been developed.

The group of Dr. Rosell in Spain has developed and validated a method for *EGFR* mutation testing in samples containing less than 150 tumor cells that can be applied to both fresh and paraffin-embedded biopsies and cytologic specimens. Their method is based on micro-dissection of tumor cells directly into PCR buffer, followed by amplification, and determination of *EGFR* status by fragment length analysis of fluorescently-labeled products (exon 19 deletions) or TaqMan Assay (exon 20 T790M and exon 21 L858R). Its spectrum is restricted to mutations in exon 19 and the L858R in exon 21, which account for the large majority of sensitive *EGFR* mutations [19].

The Therascreen EGFR RGQ PCR kit (Qiagen) is a commercial CE-labeled kit for the diagnostic analysis of *EGFR* mutation status. The assay is based on allele-specific amplification, applying the technology of the Amplification Refractory Mutation System (ARMS) [20, 21]. The amplification is followed in real-time by Scorpion technology [22, 23]. The kit allows the detection of a wider scope of mutations, including 19 deletions of exon 19, three insertions in exon 20, the point mutations G719A/S/C (exon 18), S768I, and T790M (exon 20) and L858R and L861Q (exon 21). In addition to its better detection threshold, the kit is less time consuming than Sanger sequencing [18]. The Therascreen assay does however not yield sequence information about the type of codon 719 changes, the exon 19 deletions, or the exon 20 insertions. The cost of this assay is also substantially higher than the cost of Sanger sequencing.

The goal of this study was to extend the spectrum of the Rosell methodology. To this end, we evaluated a LNA based PCR/sequencing method to detect G719A/S/C mutations, exon 19 deletions and exon 20 insertions. Requirements were a detection threshold comparable to that of commercial kits, and a cost lower than commercial kits. For convenience, applicability of a single platform was considered an asset. We developed a wild-type blocking PCR by addition of a highly sensitive locked nucleic acid (LNA) probe complementary to the wild-type sequence. LNA are bicyclic nucleic acids where a ribonucleoside is linked between the 2’-oxygen and the 4’–carbon atoms with a methylene unit. For each incorporated LNA monomer, the melting temperature (*T*m) of the duplex increases by 2–8 °C. As a result, LNA probes exhibit unprecedented thermal stability when hybridized to a complementary DNA or RNA strand, and substitutions of DNA nucleotides with LNA allow short probes while maintaining a high *T*m. The use of LNA probes complementary to the wild-type sequence, effectively suppresses amplification of the wild-type allele and leads to preferential amplification of the mutant allele [24].

Here, we evaluate the development of a LNA-PCR/sequencing assay for codon G719 (exon 18), exon 19 deletions, and exon 20 insertions.

## Methods

### Tumor samples

Our institutional *EGFR* testing database includes patients diagnosed at the University Hospitals Leuven with advanced stage NSCLC, subtyped as adenocarcinoma, NSCLC-not otherwise specified (NOS) or squamous cell carcinoma with a negative/light smoking history. For the LNA study, we included diagnostic tissue specimens obtained by bronchoscopy (bronchial biopsies and endobronchial ultrasound guided transbronchial needle aspirations (EBUS-TBNA)) between September 2010 and December 2012. For the validation study, we used archived surgical resection specimens from 31 early stage lung adenocarcinoma patients.

### DNA extraction

For *EGFR* mutation analysis on small biopsies and cytology specimens, twelve consecutive 4 μm sections were prepared from the paraffin block, the first, and last of which were stained with H&E, and evaluated for the presence and amount of tumor cells by an experienced pathologist. The proportion of tumor cells was estimated semi-quantitatively and the representative area was marked on the H&E slide. After deparaffination with xylene and alcohol, the tumor foci in the marked areas were manually macro-dissected, using a mini lambotte KA 480/04 (Devroe Instruments, Londerzeel, Belgium), and collected in microtubes. The tissue was digested overnight with proteinase K. DNA extraction was done using the Maxwell16 FFPE tissue LEV DNA purification kit on the Maxwell16 instrument (Promega, Madison, WI) according to manufacturer’s instructions. The concentration of the extracted DNA was measured using Victor spectrophotometry (Perkin Elmer, Waltham, MA). For surgical biopsies in the validation study, one H&E slide followed by 5 serial unstained sections (4 μm thick) and a final H&E were prepared and both H&E sections were evaluated. DNA extraction was done using the QIAamp DNA FFPE Tissue Kit (Qiagen, Valencia, CA) according to the manufacturer’s instructions. DNA was quantified by the Quant-iT PicoGreen dsDNA Assay Kit (Invitrogen by Life Technologies, Grand Island, NY).

### LNA-PCR

The LNA suppresses the amplification of the wild-type allele, leading to preferential amplification of mutant sequences. The real-time amplification was done on a LightCycler 480 II system (Roche Applied Science, Indianapolis, IN). SybrGreen I was used for the detection of PCR products. Each specimen was subjected to two reactions, one without LNA, and one with LNA. Primers and LNA probe sequences are listed in Table 1. Diagnostic samples with known *EGFR* mutation status as determined with the Therascreen EGFR RGQ PCR kit were used as test material. Fifty ng of input DNA was used. Each qPCR reaction was performed in a sealed LightCycler 480 Multiwell Plate 96 (Roche) in a 25 μl volume mixture containing 50 ng of genomic DNA, 6.25 μl LightCycler 480 SYBR Green I Master (Roche), 6.25 μl TaqMan Universal PCR Master Mix (Applied Biosystems by Life Technologies, Grand Island, NY), and 0.12 μM forward and reverse primer (= control reaction). In the LNA reaction, 0.6 μM of the LNA probe was added to the PCR mixture. The PCR amplification was carried out as follows: 10 minutes at 95 °C and 45 cycles of 30 sec. at 95 °C and 45 sec. at 62 °C. LightCycler 480 software release 1.5.0 was used for mutation identification by Advanced Relative Quantification Analysis. Sample delta crossing point (∆Cp)-values were calculated as the difference between the Cp-value of the LNA reaction and the Cp of the control reaction. Reactions with a ∆Cp less than the cut-off value were considered positive, i.e. potentially indicating an *EGFR* mutant, and the LNA-PCR products of these cases were subjected to Sanger sequencing for confirmation.

**Table 1:** Primers and LNA probes for LNA assay with Sanger sequencing confirmation

Exon	Name	Target	Amplicon size	Sequence (5’–3’)
18	18 FP	G719A/S/C	112 bp	*CAGGAAACAGCTATGACC*TGGAGCCTCTTACACCCAGT
	18 RP			ACCGTGCCGAACGCACCGGA
	18 LNA			GAGCCCAGC
19	19 FP	Deletions	176 bp	GTTAAAATTCCCGTCGCTATCA
	19 RP			*TGTAAAACGACGGCCAGT*TGTGGAGATGAGCAGGGTCT
	19 LNA			CAAGGAATTAAGA
20	20 FP	Insertions	161 bp	GAAGCCTACGTGATGGCCA
	20 RP			*CAGGAAACAGCTATGACC*AGCAGGTACTGGGAGCCAAT
	20 LNA			CAGCGTGGACA
-	M13-tag 1	For Sanger sequencing	-	TGTAAAACGACGGCCAGT
-	M13-tag 2	For Sanger sequencing	-	CAGGAAACAGCTATGACC

*Italic:* M13-sequencing tag; *F:* forward primer; *R:* reverse primer; *LNA:* locked nucleic acid; *bp:* base pairs

In a theoretical situation with 100 % mutant tumor, the ∆Cp is expected to be zero. At 10 % mutant, the expected ∆Cp-value is 3.32 (^2^log10 = 3.32). At 1 % mutant, the expected ∆Cp will be 6.64 (^2^log100 = 6.64). Based on this theoretical assumption, we performed Sanger sequencing on cases with a ∆Cp less than 6.6.

### Sanger sequencing

Use of PCR primers containing M13-tags allows genotyping of mutations directly by sequencing without a need for additional PCR reactions. The PCR product was purified by ExoSAP-IT treatment (Affymetrix, Santa Clara, CA). Sanger sequencing of LNA-PCR products was performed using M13–sequencing primer tag 2 for exon 18, M13–sequencing primer tag 1 for exon 19 (Table 1) and the BigDye Terminator v3.1 Cycle Sequencing Kit (Applied Biosystems) on a ABI Prism 3100 Genetic Analyzer (Applied Biosystems). Sequencing was done in one direction only because of binding of the LNA probe to either the 5’ or 3’ end of the wild-type amplicon. Results were compared with the *EGFR* wild-type sequence [GenBank:NM_005228].

### EGFR mutation testing with Therascreen EGFR RGQ PCR kit

Mutation analysis of EGFR using the Therascreen EGFR RGQ PCR kit (Qiagen, Valencia, CA) was performed on the LightCycler 480 II instrument (Roche Applied Science, Indianapolis, IN). qPCR reactions were carried out in a total volume of 25 μl (19.5 μl reaction mix + 0.5 μl Taq DNA polymerase + 5 μl sample) and the following cycle conditions: 95 °C for 15 min, 45 cycles at 95 °C for 30 sec and 60 °C for 1 min. 50 ng of genomic DNA was used.

### Fragment Length Analysis

For PCR amplification of *EGFR* exon 19, the following primers were used: forward 5’–ACTCTGGATCCCAGAAGGTGAG–3’ and reverse 5’–FAM-CCACACAGCAAAGCAGAAACTC–3’. PCR was performed in 25 μl volumes adding 5 ng of DNA, 2.5 μl GeneAmp Gold Buffer, 200 μM dNTPs, 1.5 mM MgCl2, 0.625 U AmpliTaqGold DNA Polymerase (Applied Biosystems by Life Technologies, Grand Island, NY), and 0.5 μM of each primer. Amplification was done for 32 cycles (30 seconds at 95 °C, 30 seconds at 61 °C, and 1 minute at 72 °C). 0.2 μl of PCR-product was mixed with 0.5 μl of Rox size standard (Applied Biosystems) and denatured in 20 μl HiDi formamide (Applied Biosystems) at 95 °C for 3 minutes. Separation was done with a four-color laser-induced fluorescence capillary electrophoresis system (ABI Prism 3100 Genetic Analyzer, Applied Biosystems). The collected data were evaluated with the ABI Prism GeneScan and Genotyper software.

## Results

We developed an assay for *EGFR* mutations G719A/S/C in exon 18 and deletions in exon 19 based on a LNA wild-type blocking PCR followed by Sanger sequencing of the amplicon.

Diagnostic samples with known *EGFR* status (wild-type or mutant) were used as test materials. The samples are listed in Table 2 with their ΔCp and *EGFR* status. The ΔCp of wild-type samples was comparable with the ΔCp of the CCL221 cell line, used as negative control and far above the cut-off value of 6.6. We had two cases with G719 mutations (exon 18). They had a ΔCp-value of 2.3 and 1.4. Subsequent Sanger sequencing of the LNA-PCR product indicated a p.G719C mutation for sample L62 and a p.G719A mutation for sample L77. We analyzed five cases with exon 19 deletions. The ΔCp for these samples was between 1.4 and 5.6. The exon 19 deletion was confirmed by Sanger sequencing in all five samples. All mutated samples had a deletion of fifteen nucleotides, corresponding with the ELREA amino acid string.

**Table 2:** Comparison between LNA assay and Sanger sequencing

Sample	% tumor	∆Cp G719A/S/C	LNA G719A/S/C	Sanger sequencing
CCL221		10.4	negative	n.d
L86	65	10.6	negative	n.d
L18	30	10.4	negative	n.d
L30	15	10.4	negative	n.d
L62	20	2.3	positive ?	p.G719C (c.2155G > T)
L77	50	1.4	positive ?	p.G719A (c.2156G > C)
Sample	% tumor	∆Cp deletions 19	LNA deletions 19	Sanger sequencing
CCL221		10.8	negative	n.d
L86	65	10.4	negative	wild-type
L18	30	10.1	negative	n.d
L95	10	5.6	positive ?	p.E746_A750del (c.2235_2249del15)
L91	100	1.4	positive ?	p.E746_A750del (c.2236_2250del15)
L93	50	3.6	positive ?	p.E746_A750del (c.2235_2249del15)
L58	10	2.9	positive ?	p.E746_A750del (c.2235_2249del15)
L55	70	1.8	positive ?	p.E746_A750del (c.2235_2249del15)

*Cp:* crossing point; *LNA:* locked nucleic acid; *n.d:* not done

We performed dilution experiments to evaluate the detection threshold of the LNA-PCR/sequencing assay. A histological specimen consisting of approximately 100 % nucleated tumor cells and harboring a known exon 19 deletion was selected. The tumor-derived DNA was mixed with DNA from *EGFR* wild-type patients in ratios ranging from high to low tumor content (100 %–0 % tumor, serially diluted one in two times). Reactions were performed as described previously and carried out in duplicate. The theoretically chosen ΔCp cut-off value of 6.6 corresponded with an estimated malignant cell fraction of 3.125 %, while a tumor cell content of 1.5625 % yielded 6.75 as ∆Cp (Table 3).

**Table 3:** Dilution experiment LNA assay for EGFR exon 19 deletions

Sample	Cp controle	Cp LNA	∆Cp
100 % mutant	29.66	30.93	1.27
50 % mutant	29.89	31.91	2.02
25 % mutant	30.13	33.37	3.24
12.5 % mutant	30.12	33.92	3.80
6.25 % mutant	30.35	35.78	5.43
3.125 % mutant	30.56	36.34	5.78
*1.5625 % mutant*	*30.21*	*36.96*	*6.75*
*100 % wild-type*	*29.93*	*39.70*	*9.77*

*Cp:* crossing point; *LNA:* locked nucleic acid

We also attempted to design a LNA-PCR assay for the insertions in exon 20. However, the ∆Cp was between 6.6 and 6.8 for wild-type samples and between 4.2 and 10.0 for the three mutant samples tested. To resolve this discrepancy, we subjected the latter mutant samples to direct Sanger sequencing. This revealed the following mutation types: p.V769_D770insASV (duplication, ∆Cp: 4.2), D770_N771insGL (random nucleotide insertion, ∆Cp: 6.1), and p.H773_V774insNPH (duplication, ∆Cp: 10.0). The LNA used for exon 20 insertions binds to nucleotides c.2301 to c.2311 (i.e. the last nucleotide of codon 767 to the first nucleotide of codon 771 inclusive). Thus binding of this LNA is not affected by the p.H773_V774insNPH, explaining the high ∆Cp in this case. In the two other cases, there is a mismatch of one nucleotide between the targeted mutant sequence and the LNA sequence which is predicted to interfere with LNA binding, thus explaining the lower delta CP.

In conclusion the G719A/S/C in exon 18 and deletions in exon 19 were eligible for a LNA-PCR/sequencing assay. Due to technical reasons related to exon 20 insertions, a LNA-PCR testing method for this type of mutations is less straightforward.

## Validation study

We analyzed the mutation status of 31 resection specimens with the following methods: LNA-PCR with Sanger sequencing confirmation, Fragment Length Analysis, and the Therascreen EGFR RGQ PCR kit. First, the *EGFR* status of the resection specimens was determined by the LNA-PCR/sequencing testing method comprising exon 18 and 19. In this series of 31 specimens, no cases with G719A/S/C mutations were identified. The Advanced Relative Quantification Analysis of the LightCycler 480 software release 1.5.0 yielded ΔCp-values between 6.1 and 8.4 for the G719A/S/C LNA-PCR (Table 4). Sample ID29 was considered as a failure because the control Cp-value was 40. Sample ID10 had a ΔCp of 3.2. However, sequencing of the product of the LNA reaction of sample ID10 revealed gga for codon 719, a silent mutation translated to glycine (G). Due to the design of the LNA probe to block ggc, amplification of the gga allele was not blocked. Sample ID30 had a ∆Cp of only 5.4, but the control Cp-value of 34.7 indicated limited amounts of good quality DNA. Because the validation cohort did not harbor any G719 mutations, we included an additional case with a positive G719 mutation, identified by a Therascreen testing of an EBUS specimen. The ∆Cp-value of 2.1 indicated a positive reaction. Sanger sequencing of the LNA-PCR product confirmed a p.G719C mutation (c.2155G > T). This case, together with the silent mutation provide proof-of-principle that the wild-type blocking PCR detects codon 719 mutations, but more samples are required to determine sensitivity and specificity of the test for codon 719.

**Table 4:** LNA-PCR/sequencing assay on resection specimens for validation study + comparison of methods

Sample	∆Cp G719	Result LNA/ sequencing	∆Cp deletions 19	Result LNA/sequencing	Therascreen^*^	Fragment Length Analysis
ID1	6.1	neg. / n.d	7.7	neg. / n.d	wild-type	wild-type
ID2	6.8	neg. / n.d	1.3	pos. ? / n.d	exon 19 deletion	exon 19 deletion (15 bp)
ID3	7.3	neg. / n.d	7.2	neg. / n.d	wild-type	wild-type
ID4	6.7	neg. / n.d	8.4	neg. / n.d	wild-type	wild-type
ID5	7.2	neg. / n.d	8.0	neg. / n.d	wild-type	wild-type
ID6	7.2	neg. / n.d	6.5	neg. / n.d	wild-type	wild-type
ID7	7.1	neg. / n.d	7.5	neg. / n.d	wild-type	wild-type
ID8	7.4	neg. / n.d	7.3	neg. / n.d	wild-type	wild-type
ID9	7.1	neg. / n.d	8.6	neg. / n.d	wild-type	wild-type
ID10	3.2	pos. ? / wild-type	6.6	neg. / wild-type	wild-type	wild-type
ID11	7.1	neg. / n.d	7.0	neg. / wild-type	wild-type	wild-type
ID12	7.1	neg. / n.d	7.3	neg. / n.d	wild-type	wild-type
ID13	7.5	neg. / n.d	1.7	pos. ? / p.E746_A750del c.2235_2249del15	exon 19 deletion	exon 19 deletion (15 bp)
ID14	6.6	neg. / wild-type	9.2	neg. / n.d	wild-type	wild-type
ID15	7.0	neg. / n.d	7.2	neg. / n.d	wild-type	wild-type
ID16	7.3	neg. / n.d	0.3	pos. ? / n.d	exon 19 deletion	exon 19 deletion (18 bp)
ID17	7.5	neg. / n.d	7.2	neg. / n.d	wild-type	wild-type
ID18	6.8	neg. / n.d	8.5	neg. / n.d	wild-type	wild-type
ID19	6.8	neg. / n.d	1.8	pos. ? / p.E746_A750del c.2235_2249del15	exon 19 deletion	exon 19 deletion (15 bp)
ID20	6.8	neg. / n.d	5.2	pos. ? / wild-type	wild-type	wild-type
ID21	6.9	neg. / n.d	0.4	pos. ? / p.L747_P753 > S c.2240_2257del18	exon 19 deletion	exon 19 deletion (18 bp)
ID22	7.3	neg. / n.d	6.1	neg. / wild-type	wild-type	wild-type
ID23	7.5	neg. / n.d	8.2	neg. / n.d	wild-type	wild-type
ID24	7.3	neg. / n.d	8.9	neg. / n.d	wild-type	wild-type
ID25	6.9	neg. / n.d	4.9	pos. ? / wild-type	wild-type	wild-type
ID26	6.9	neg. / n.d	7.0	neg. / n.d	wild-type	wild-type
ID27	8.4	neg. / n.d	2.7	pos. ? / p.E746_A750del c.2235_2249del15	exon 19 deletion	exon 19 deletion (15 bp)
ID28	7.5	neg. / n.d	7.5	neg. / n.d	wild-type	wild-type
ID29	/	/	/	/	/	wild-type
ID30	5.4	pos. ? / wild-type	1.0	pos. ? / wild-type	/	wild-type
ID31	7.7	neg. / n.d	8.9	neg. / n.d	wild-type	wild-type

^*:^G719X reaction mix and deletions 19 reaction mix; *Cp*: crossing point; *LNA*: locked nucleic acid; *del*: deletion; *bp*: base pairs; *neg.*: negative result; *n.d*: not done; *pos.*: positive result

An exon 19 deletion mutation was found in six of the 31 resection specimens, with ΔCp-values ranging between 0.3 and 1.8. Sanger sequencing of the LNA products confirmed these deletions (Table 4). The ΔCp-values for the samples with a wild-type status varied between 6.1 to 9.2 with three exceptions. Sample ID20 had a ∆Cp of 5.2 but had a higher Cp value of the control reaction (34.8). Samples ID25 and ID30 had also a higher control Cp value (35.2 and 39.0). Sequencing data of these three samples confirmed the absence of mutations. Again the control Cp-value of sample ID29 was above 40 and considered as technical failure due to poor DNA quality.

In the second step of the validation study the 31 resection specimens were subjected to Fragment Length Analysis (exon 19) and to the Therascreen EGFR RGQ PCR kit (exon 18 and 19). The results were summarized in Table 4. Sample ID29 and ID30 were excluded due to poor DNA quality as seen before in the LNA-PCR/sequencing testing method.

The *EGFR* mutation status obtained with our LNA-PCR/sequencing assay therefore correlated adequately with the results obtained by the other methods.

## Discussion

Activating mutations of *EGFR* observed in NSCLC occur in the intracellular tyrosine kinase domain, i.e. within exons 18–21 of the receptor [2, 3]. The most common *EGFR* mutations are small in-frame deletions in exon 19 and the L858R point mutation in exon 21 [4, 5]. They account for about 90 % of all *EGFR* mutations reported in lung adenocarcinoma and are known to be sensitive to the EGFR-TKIs gefitinib, erlotinib, or afatinib [7–10]. A high response rate to these TKIs was also seen in patients with a somatic mutation in exon 18 (G719A/S/C), which occurs with a frequency of approximately 3 % [5, 7–9]. By contrast, the T790M mutation in exon 20 is associated with acquired resistance to TKIs [11, 12] and the insertions in exon 20 are less sensitive to *EGFR*-TKIs compared with other sensitizing mutations [13, 14].

Treatment decisions in advanced stage rely on accurate analysis of the *EGFR* mutation status in small tissue samples obtained by bronchoscopic procedures [16, 17]. However, mutation analysis on small FFPE tissue samples faces several challenges. Some are sample related, including limited amounts of sample material, low tumor cell content, and suboptimal DNA quality. Other concerns are related to the performance characteristics of the technology used for mutation analysis, e.g. the detection threshold, the mutation spectrum, the cost of the consumables and the hands-on time [25].

We present a wild-type blocking LNA-PCR amplification assay followed by confirmation by sequencing of amplification product of positive reactions. The assay determines the *EGFR* mutation status of codon G719 (exon 18) and exon 19 deletions and can be an alternative for commercially available kits, as codon G719 mutations were not in the scope of previously published non-commercial methodologies [19]. We have validated results for exon 18 and for exon 19. Based on a theoretical assumption, the ∆Cp cut-off value was initially set at 6.6: specimens with a lower ∆Cp were considered as potentially indicative of a mutation and the amplification product was submitted to Sanger sequencing for confirmation. This theoretical ∆Cp threshold chosen corresponded with a threshold of 3.125 % infiltration by malignant cells under experimental conditions. This difference with the theoretical calculation is likely due to PCR efficiency less than 100 %. However this detection threshold is better than conventional Sanger sequencing, competitive with the Therascreen assay, and therefore adequate for clinical testing.

Exon 20 insertions and duplications are less suited for wild-type blocking approaches. This is due to the molecular heterogeneity of exon 20 *EGFR* mutations (Catalogue of Somatic Mutations, http://cancer.sanger.ac.uk/cosmic), as we have also shown in our series. This known heterogeneity of insertions/duplications would require the design of several LNA probes, defeating the purpose of a simple wild-type blocking strategy to screen for exon 20 insertions. To our knowledge, there are no published reports on LNA technology for exon 20 insertions. Other techniques such as fragment length analysis are more appropriate, if one wishes to test for this class of *EGFR* mutations.

We also found a ∆Cp less than 6.6 in three specimens with low amounts/quality of extracted DNA, which were not confirmed by Sanger sequencing. Larger validation series should be investigated in order to define the optimal ∆Cp as well as control Cp value cut-offs, which compromise best between sensitivity and specificity. For the time being, we consider it safest not to reject samples with high control Cp values, as the final results depend on confirmation by sequencing of the generated amplicon product anyway.

The combination of allele-specific PCR reactions for the T790M (exon 20) and L858R (exon 21) point mutations with this assay, has a wider scope of *EGFR* mutations. Introduction of minor modifications of Rosell’s TaqMan assay for T790M and L858R, allows to adapt the latter assays to the LightCycler 480 II instrument. Cost calculation indicated that the reagent cost for these tests on a LightCycler platform is only about 25 % compared with the Therascreen EGFR RGQ PCR kit (based on list prices). However, these assays require slightly more hands-on time, due to the need for confirmation of positive cases, which results in a higher personnel cost. Overall, the cost remains 25 to 50 % lower in comparison with the Therascreen testing method.

## Conclusions

In conclusion, we propose an alternative methodology, to determine the *EGFR* mutation status for G719 (exon 18) and exon 19 deletions in NSCLC patients in clinical routine. The LNA-PCR/sequencing assay needs less input DNA, is specific, sensitive, and has a detection threshold of 3.125 % malignant cells. In combination with allele-specific PCR reactions for T790M (exon 20) and L858R (exon 21), this methodology allows to assess a wider scope of clinically relevant mutations, provides sequence information, and runs at a lower cost than available commercial kits.

## Abbreviations

NSCLC: non-small cell lung cancer
EGFR: epidermal growth factor receptor
TKI: tyrosine kinase inhibitor
qPCR: quantitative real-time PCR
ARMS: amplification refractory mutation system
LNA: locked nucleic acid
*T*m: melting temperature
NOS: not otherwise specified
EBUS-TBNA: endobronchial ultrasound guided transbronchial needle aspiration
Cp: crossing point
